# Thermal Influence of CNT on the Polyamide 12 Nanocomposite for Selective Laser Sintering

**DOI:** 10.3390/molecules201019041

**Published:** 2015-10-20

**Authors:** Jiaming Bai, Ruth D. Goodridge, Shangqin Yuan, Kun Zhou, Chee Kai Chua, Jun Wei

**Affiliations:** 1 Singapore Institute of Manufacturing Technology, 71 Nanyang Drive, 638075 Singapore, Singapore; E-Mails: yuan0057@e.ntu.edu.sg (S.Y.); jwei@simtech.a-star.edu.sg (J.W.); 2 Additive Manufacturing and 3D-Printing Research Group, Faculty of Engineering, University of Nottingham, Nottingham NG7 2RD, UK; E-Mail: Ruth.Goodridge@nottingham.ac.uk; 3 School of Mechanical and Aerospace Engineering, Nanyang Technological University, 639798 Singapore, Singapore; E-Mails: kzhou@ntu.edu.sg (K.Z.); mckchua@ntu.edu.sg (C.K.C.)

**Keywords:** selective laser sintering, thermal influence, numerical simulation, polymer nanocomposites

## Abstract

The thermal influence of carbon nanotubes (CNTs) on the PA12 in the laser sintering process was assessed by physical experiments and a three dimensional simulation model. It appears that, by adding the CNTs into the PA12 matrix, the thermal conductivity increased. A double ellipsoidal heat flux model was applied to input a three dimensional, continuous moving, volumetric laser heat source. The predicted three dimensional temperature distributions suggested that the laser heat was conducted wider and deeper in the PA12-CNT sample than PA12. Greater heat conduction can reduce the interspace between two successive layers, and result in the increase of the parts’ density and properties.

## 1. Introduction

Additive manufacturing (AM) is a collection of advanced manufacturing processes which produce physical 3D objects directly from 3D model data [1]. Often referred to as 3D printing nowadays, these processes build a finished part layer-by-layer in an additive way, as opposed to the conventional subtractive processes that cut away material from a big work piece, or molding manufacturing processes [2]. AM, which is an outgrowth of rapid prototyping, aims to offer greater flexibility in respect to product design and manufacture compared to traditional manufacturing routes.

Laser sintering, or selective laser sintering, is one of the most established powder-based AM process. One of the advantages of laser sintering compared to other AM techniques such as fused deposition molding or selective laser melting, is that no support structure are required as the surrounding un-fused powder provides support, which avoids some post-processing and reduces manpower. There are in theory large numbers of polymer materials in powder form that can be processed using laser sintering. However, in reality very few polymers are currently available for laser sintering compared with the hundreds to thousands of grades available for conventional manufacturing techniques, such as injection molding. Furthermore, the polymers that are available at the moment cannot completely meet the needs of all applications due to unsatisfactory mechanical, thermal or electrical properties [3].

Due to the current limited choice of single materials for additive manufacturing and laser sintering, polymer/filler composites have been investigated to enhance the mechanical, thermal, or electrical properties [4,5,6,7,8]. In the work carried out by Goodridge *et al.* [9], a 3 wt % PA12/carbon nanofiber composite was prepared by melt mixing and cryogenic milling. The dynamic mechanical properties of the nanocomposite were 22% higher than those of the base PA12 laser sintered parts. In the recent study, a novel method to prepare PA12-carbon nanotube (CNT) nanocomposite powder by coating the CNTs onto the surface of PA12 powder particles was used [10,11]. This allowed the optimized size and near-spherical morphology of the commercial laser-sintering PA12 powder to be retained. Results showed that the PA12-CNT laser sintered parts had enhanced flexural properties and impact properties [4].

Laser sintering is a complex thermal process which contains heat absorption, heat transfer and phase change. Thermal properties of the laser sintering materials are important in understanding the process-properties relationships. As there is currently no efficient experimental method to examine the thermal phenomenon during the laser sintering process, computer simulation is very important in understanding the laser sintering process [12,13]. In the work carried out by Nelson *et al.* [14], a one-dimensional heat transfer model was developed to predict the sintering depths and density of laser sintered polycarbonate. Tontowi and Childs [15] developed a two-dimensional finite element model for semi-crystalline polymers, which included the latent heat of melting, and allowed for the CO_2_ laser radiation to be absorbed over a finite depth of the powder bed. However, the thermal effect of the nanofiller on the SLS process has rarely been reported. In this work, experimental and simulation investigation of a polymer nanocomposite for laser sintering was carried out to examine the thermal influence of the nanofiller on the polymer matrix during laser sintering process.

## 2. Experimental Approach

### 2.1. Materials

The base polymer selected for this study was PA2200 (EOS GmbH, Krailling, Germany), a PA12 material, which has an established use in the laser sintering process. Multiwalled CNTs were supplied by NanoAmor Materials Inc., Houston, TX, USA, which had an average diameter of 10 nm and length of 1.5 µm according to the manufacturing datasheet. The PA12-CNT nanocomposites were prepared by coating the CNTs on to the surface of the PA12 powder particles, with a loading of 0.1 wt % [4]. The final PA12-CNT nanocomposites powders have been shown to exhibit near-spherical morphology with the CNT uniformly coated on the surface. The PA12-CNT nanocomposites powders, which had an average particle size of 57 µm, were laser sintered on an EOS P100 laser sintering machine (EOS GmbH) with optimum processing parameters.

### 2.2. Density

To determine the powder bed density ρ, density cube method was used [16]. Firstly, PA12 and PA12-CNT hollow cubes were produced by laser sintering with precise geometry. After laser sintering, the mass of the un-sintered powders left in the hollow cube can be measured. Then, with the known hollow volume, the density of the un-sintered powders can be calculated, which was used as the powder density during laser sintering.

### 2.3. Thermal Conductivity 

Thermal conductivities of the PA12 and PA12-CNT were measured by a thermal conductivity apparatus (Cussons Technology Ltd., Salford, UK). The apparatus consists of a vertical stack of specimens clamped between an electrically heated source at the top and a water cooled base, all located within a Dewar vessel and furnished with a radiation shield and anti-convection baffle.

**Figure 1:**
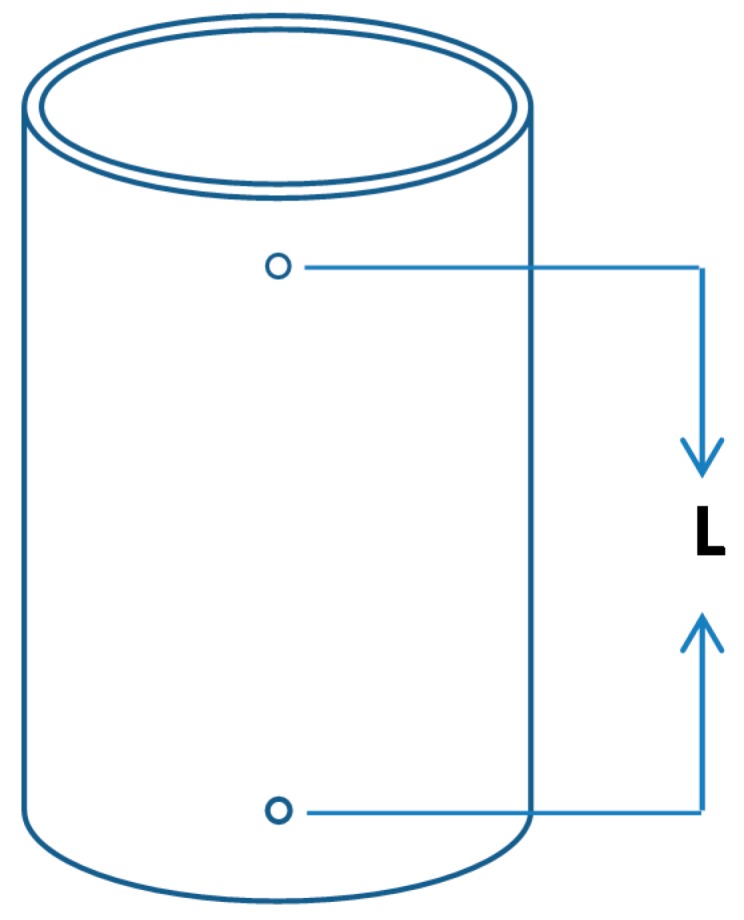
Schematic of the hollow cylinder specimen made by laser sintering.

The sample specimen was designed as a hollow cylinder (height = 38 mm, diameter = 20mm, wall thickness = 0.5 mm) and produced by laser sintering, in which the testing materials were stored, shown in Figure 1. In this work, testing temperature was chosen from 100–175 °C, with an interval of 25 °C.

The thermal conductivity is calculated as:

(1) $$K=\;\frac{C_{P}\times M\times L\times \left(W_{2}-W_{1}\right)\;}{A\times t\times \left(T_{4}-T_{3}\right)}$$

where *C_P_* is the specific heat capacity of water (4186 Joules/Kg), *M* is the mass of water collected in a certain time (*t*), *L* is the distance between the two thermocouples (*T*_1_ and *T*_2_), *W*_1_ is the water inlet temperature, *W*_2_ is the water outlet temperature, *A* is the cross-section area of the specimen, *t* is the time for collection of *M*, *T*_1_ to *T*_4_ are the thermocouple temperatures.

**Figure 2:**
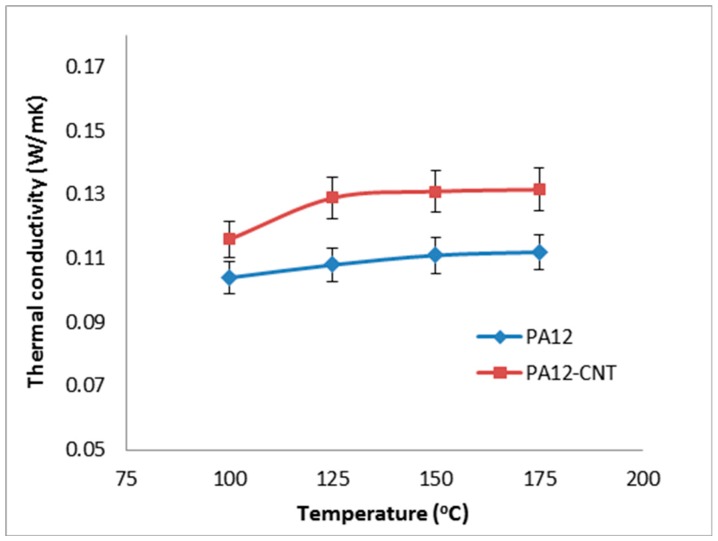
Thermal conductivity of PA12 and PA12-carbon nanotube (CNT) powders.

The results of the thermal conductivity of the PA12 and PA12-CNT are shown in Figure 2. PA12-CNT appeared average 14.2% greater thermal conductivity than that of PA12. It can be seen that as the temperature rise, the thermal conductivity for both PA12 and PA12-CNT increased very slightly. It was suggested that for polyamide, a constant thermal conductivity could be used as the thermal conductivity is not sensitive to temperature. Therefore, in the current research, thermal conductivity was assumed temperature-independent and isotropic.

### 2.4. Specific Heat

The specific heat of both PA12 and PA12-CNT were determined by DSC method [17]. With ~20 mg sample, the heating rate of this test was set to 10 °C/min. By incorporating the correction factor, the specific heat of the polymers was calculated from the DSC curve. In the model developed here, the specific heat is presumed to be independent of temperature.

The summary of material properties used in this model is listed in Table 1.

**Table 1:** Materials properties of PA12 and PA12-CNT used in the simulation.

Property	PA12	PA12-CNT
Density (ρ), kg/m^3^	445.0 (±0.1)	445.4 (±0.1)
Thermal conductivity (k), W/(mK)	0.111 (±0.003)	0.131 (±0.005)
Specific heat (C_p_), J/(kgK)	2660.0 (±2.5)	2500.0 (±5.5)
Glass transition temperature (T_g_), °C	58.5 (±0.1)	58.5 (±0.1)
Melting temperature (T_m_), °C [4]	183.4 (±0.2)	184.0 (±0.2)

## 3. Modelling Approach

### 3.1. Materials Properties

The main materials properties used herein for the laser sintering heat transfer simulation are: medium’s density ρ, thermal conductivity λ and specific heat *C*. A master equation of three-dimensional heat transfer within a material can be presented as:

(2) $$\text{ρ}C\frac{\partial T}{\partial t}=\;\frac{\partial }{\partial x}\left(\text{λ}\frac{\partial T}{\partial x}\right)+\;\frac{\partial }{\partial y}\left(\text{λ}\frac{\partial T}{\partial y}\right)+\;\frac{\partial }{\partial z}\left(\text{λ}\frac{\partial T}{\partial z}\right)+\text{ Φ}$$

where *t* is the time, *T* is the temperature, *x*, *y*, *z* are the coordinates and Φ is the heat from laser scanning. 

As the main purpose of this simulation is to compare the thermal process of PA12 and PA12-CNT during laser sintering, simplifying assumptions are made about the material properties during the laser sintering process in this work. Both PA12 and PA12-CNT are assumed continuous and homogeneous with isotropic properties, and temperature independent during the laser sintering process. Relative experiments were carried out to determine the accurate value of the properties of PA12 and PA12-CNT used herein.

### 3.2. Laser Heat Source

For a reliable thermal model, it is very important to describe the heat source accurately. Two commonly methods are used for the energy input by the laser beam. The simple method is to model the laser beam as a surface heat flux condition. A more accurate method is to assume that the laser beam heating is a volumetric heat flux where energy drops with depth, which is applied in this study.

**Figure 3:**
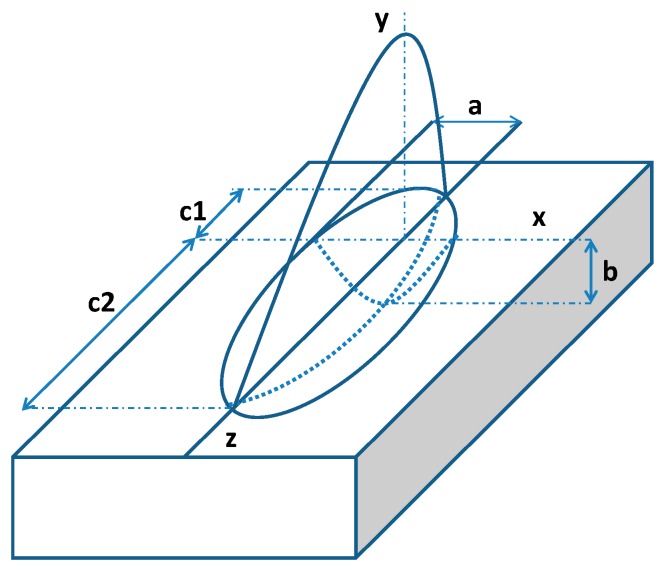
Schematic of double ellipsoidal heat source model.

As laser sintering is a high energy density process with features of intensity heating area and high penetration depth, normal Gaussian heat distribution was not chosen in this work. A double ellipsoidal volumetric heat density distribution, which is developed from general Gaussian heat distribution, was chosen as laser heat source in this model, shown in Figure 3. This heat source is a combination of two half ellipsoids—one in advance of the center of the heat source and the other at the rear, which considers the asymmetry temperature gradient of the center of the moving laser heat source.

The heat flux density inside the front ellipsoid is [18]:

(3) $$q\left(x.y.z.t\right)=\;\frac{6\sqrt{3}f_{f}Q}{abc\pi \sqrt{\pi }}\;e^{-\frac{3x^{2}}{a^{2}}\;}e^{-\frac{3y^{2}}{b^{2}}\;}e^{-\frac{-3{\left[z+v\left(\tau -t\right)\right]}^{2}}{c_{1}{}^{2}}\;}$$

Similarly the rear ellipsoid of the heat distribution becomes:

(4) $$q\left(x.y.z.t\right)=\;\frac{6\sqrt{3}f_{r}Q}{abc\pi \sqrt{\pi }}\;e^{-\frac{3x^{2}}{a^{2}}\;}e^{-\frac{3y^{2}}{b^{2}}\;}e^{-\frac{-3{\left[z+v\left(\tau -t\right)\right]}^{2}}{c_{2}{}^{2}}\;}$$

where *x*, *y*, *z* are the coordinate, *a*, *b*, *c*_1_, *c*_2_ are the heat source (melting pool) shape parameters, *Q* is the heat input, *v* is the heat source speed, *t* is time, τ is a time factor, *f_f_*, *f_r_* are two constants associated with the front and rear energy distribution, *f_f_* + *f_r_* = 2 approximately. In general calculation, *c*_1_ = *c*_2_ = *c* for simplifying purpose, therefore a governing equation can be expressed as:

(5) $$q\left(x.y.z.t\right)=\;\frac{6\sqrt{3}f_{f,r}Q}{abc\pi \sqrt{\pi }}\;e^{-\frac{3x^{2}}{a^{2}}\;}e^{-\frac{3y^{2}}{b^{2}}\;}e^{-\frac{-3{\left[z+v\left(\tau -t\right)\right]}^{2}}{c^{2}}\;}$$

In the current study, the diameter of the focused laser beam is 0.42 mm, which lead to *a* = 0.21 mm. *b* and *c* are estimated by analytical and regression methods combining with simulation and experiment data got in this model [19].

Commercial finite element analysis software ABAQUS^®^ is used in the study for modeling the laser sintering process. The double ellipsoidal heat distribution is defined by user Sub-routine DFLUX.

### 3.3. Boundary Conditions and Interactions

The environment temperature is set as a constant in this model, which is the powder bed warming up temperature during the laser sintering process. Convection and radiation thermal exchange with the environment is also considered.

During the laser sintering process, the high energy laser beam scans the materials in a very transient time. The laser energy is rapidly absorbed by the materials and converted into heat, which produces an instant high-temperature region to melt the material very rapidly. The energy obtained by the materials from the laser heat is much greater than the energy released by radiation, therefore the radiation thermal exchange is not considered for simplify in this model. Then, the equation can be rewritten as follows:

(6) $$k\frac{\partial \text{T}}{\partial \text{z}}=h\;\left(T-\;T_{env}\right)\;-q$$

In summary, the laser properties, initial conditions and boundary conditions applied in this model are outlined in Table 2. The laser sintering process was then simulated by applying the parameters in Table 1 and Table 2.

**Table 2:** Values of the laser properties, initial condition, and boundary condition.

Component	Value
Laser power (P), W	25
Laser scan speed (V), m/s	2.5
Laser scan space (H), mm	0.25
Laser beam diameter (D), mm	0.42
Environment temperature (T_env_), °C	172
Initial and semi-infinite boundary temperature (T_0_), °C	172
Convection coefficient (h), W/m^2^k	25

### 3.4. Temperature Distribution

**Figure 4:**
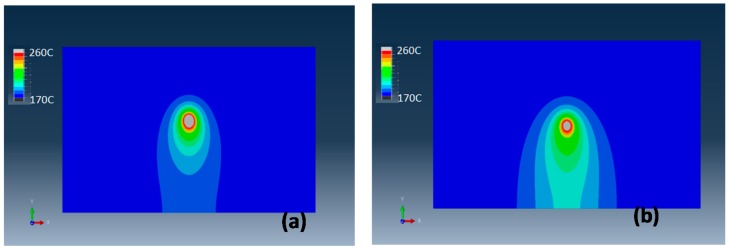
Temperature distribution of (**a**) PA12 and (**b**) PA12-CNT at *x*-*y* plane.

The temperature distribution profiles predicted by simulation at *x*-*y* plane with a moving laser beam for the PA12 and PA12-CNT are shown in Figure 4. The laser beam entered the modeled sample at the bottom side and then moved from bottom to top along the *y* axis. The temperature scales, which are listed on the left side in each figure, are identical. 

From Figure 4, it can be seen that for PA12 and PA12-CNT, the temperature range under the laser beam is very similar. The minimum value of the temperature is 172 °C (displayed as blue), which is the powder bed temperature. The maximum temperature is observed at the center of laser beam (displayed as grey), with a value about 260 °C. It can be seen that the laser beam has a concentrated heat affected area-melting pool, which showed a very stable geometry during the laser scanning process. Moving laser source with a stable and concentrated melting pool could offer desired dimensional accuracy for the sintered parts. The temperature gradient is very large close the laser spot, while the region away from the laser spot shows a gentle temperature changes. Compared to PA12, the laser beam produces a wider laser heat affecting area with PA12-CNT, which indicates that the laser heat is conducted more effectively in PA12-CNT.

It was reported that the thermal conductivity *k* of CNT is as high as over 3000 W/mK [20]; this value is remarkably higher than that of PA12 powder, which is about 0.11 W/(mK). It appears that by adding the CNTs into the PA12 powder, the heat from the laser beam has been more efficiently conducted by the powder. This could increase the fusion of the powder and flow of melted PA12, resulting in denser and stronger parts.

### 3.5. Melting Depth

**Figure 5:**
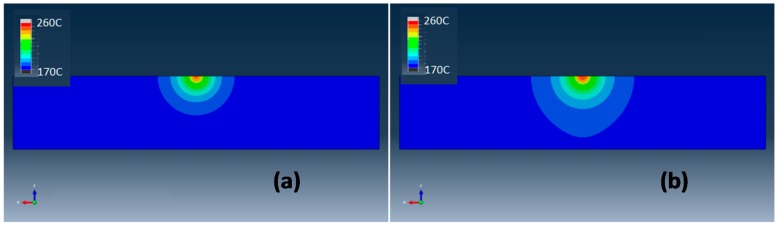
Melting depth modeling of laser sintering of (**a**) PA12 and (**b**) PA12-CNT.

One of the advantages of using double ellipsoidal heat source model is the ability to evaluate the melting depth for the laser sintering process. Figure 5 presents the predicted temperature distribution of PA12 and PA12-CNT at *x*-*z* plane. The rainbow semicircle represents the laser beam heat affected area in *z* direction.

Laser sintering is a layer-by-layer manufacturing process, where two successive layers are bonded together, therefore melting depth is very important which matters the degree of densification of the laser sintered parts. When the laser is scanning the current layer, greater melting depth could lead to a partial re-melting of the previous layer, which can reduce the interspace between these two layers and resulting in the increase in the parts’ density. It can be seen that PA12-CNT showed a larger maximum melting depth in the laser scanning process compared to PA12, which could lead to better melting in the depth direction and greater densification of the sintered parts.

**Figure 6:**
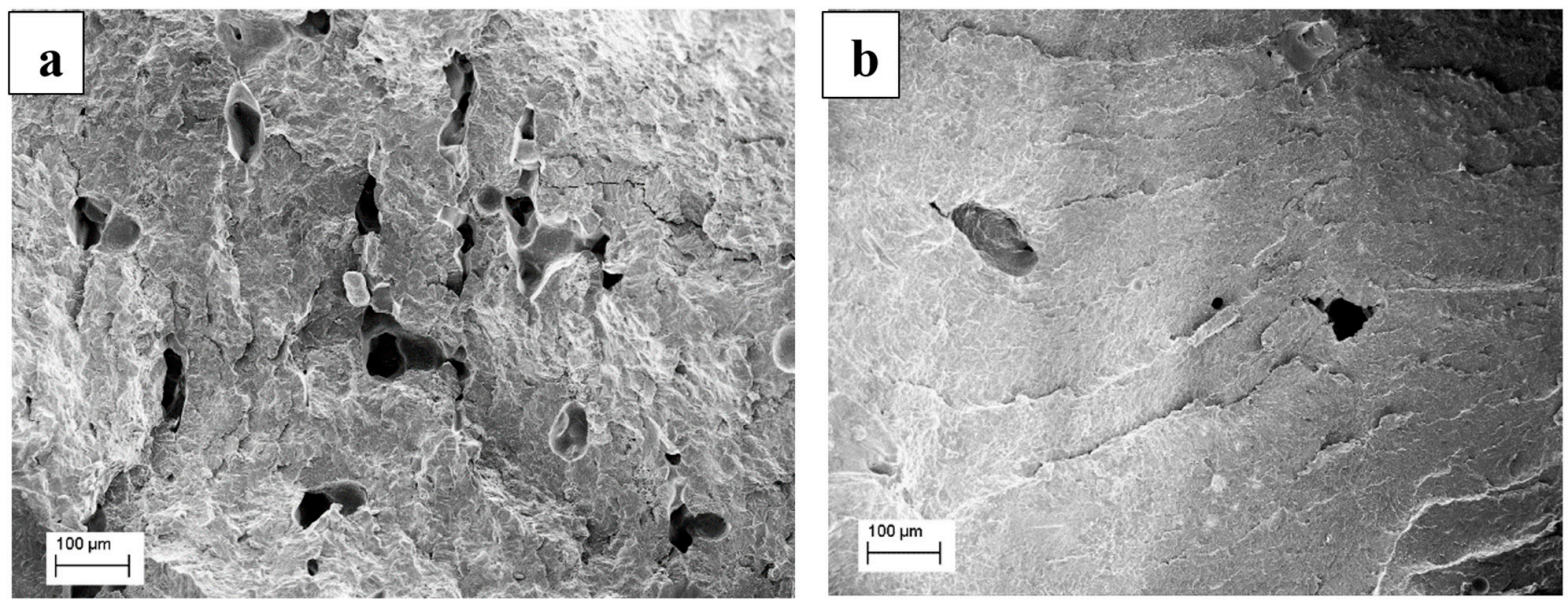
Fracture surface of (**a**) PA12 and (**b**) PA12-CNT parts.

The fracture surface of the laser sintered PA12 and PA12-CNT parts were listed in Figure 6. It can be seen that in Figure 6a, unmelted powder and voids remained in the neat PA12 sintered parts, which were not observed in the PA12-CNT parts (Figure 6b), which supports the simulation results. 

## 4. Conclusions

In this work, the thermal influence of CNT on the PA12 laser sintering process was assessed by physical experiments and three dimensional computer modeling. The heating and cooling stages of the laser sintering process were addressed with a continuous moving laser beam. A double ellipsoidal heat flux model was applied to input a three dimensional, continuous moving, volumetric laser heat source.

The predicted results of three dimensional temperature distribution suggested that the laser heat was conducted wider and deeper in the PA12-CNT compared to PA12, which was due to the greater thermal conductivity of PA12-CNT. As an advantage of using the double ellipsoidal model to simulate the laser heat flux, the melting depth was modeled herein. Simulation results illustrated that PA12-CNT sample showed a bigger melting depth under laser beam scanning compared to PA12, which could lead a denser sintered part, and this was supported by SLS experiments.
